# Body Composition and Bone Health Status of Jockeys: Current Findings, Assessment Methods and Classification Criteria

**DOI:** 10.1186/s40798-022-00414-1

**Published:** 2022-02-14

**Authors:** Arthur Dunne, Giles Warrington, Adrian McGoldrick, Jennifer Pugh, Michael Harrison, SarahJane Cullen

**Affiliations:** 1 Department of Sport and Exercise Science, Waterford Institute of Technology, Waterford, Ireland; 2 Department of Physical Education and Sport Science, University of Limerick, Limerick, Ireland; 3 Sport and Human Performance Research Centre, Health Research Institute, University of Limerick, Limerick, Ireland; 4 Irish Horseracing Regulatory Board, Kildare, Ireland

**Keywords:** Body composition, Body fat, Bone health, DXA, Horseracing, Weight making, Bone strength, Bone mineral density

## Abstract

Jockeys are unlike other weight-making athletes as the sport of horse racing requires strict weight management to meet the racing stipulations, protracted working hours and an extended racing season with limited downtime. Several studies have reported on the body composition and bone status of male and female professional and retired jockeys, yet the variety of assessment techniques, lack of standardised testing protocols and classification inconsistency make interpretation and comparison between studies problematic. This review aimed to appraise the existing body composition and bone health evidence in jockeys and evaluate the assessment methods and classification criteria used. Dual-energy X-ray absorptiometry (DXA) has been used most frequently in jockey research to assess body composition and bone status, while various generic skinfold equations have been used to predict body fat percentage. Evidence indicates flat jockeys are now taller and heavier than the data reported in earlier studies. Absolute fat mass has steadily increased in male jockeys in the last decade. The bone status of male jockeys remains a concern as constant low bone density (BMD) is evident in a large percentage of young and experienced professional jockeys. Due to limited studies and variations in assessment methods, further research is required to investigate bone turnover markers in male and female jockeys. A standardised testing protocol using internationally recognised assessment guidelines is critical for the accurate interpretation and evaluation of body composition and bone health measurements. Furthermore, establishing jockey-specific BMD and bone turnover reference ranges should be considered using existing and future data.

## Key Points

- DXA has been used most frequently in jockey research to assess body composition and bone status, while skinfold thickness and various generic skinfold prediction equations have been used to predict body fat percentage.
- Fat mass in male jockeys has increased in the last decade, thus indicating the possibility of greater difficulty to make weight and the reliance on unhealthy weight loss techniques to reach the low designated riding weights.
- Male flat jockeys present with consistently lower bone density values at reported sites than male national hunt and female jockeys. Without standardised testing protocols in jockey research, the accuracy of interpretation for bone status is limited.
- Jockey-specific classification criteria and reference ranges should be considered for the appropriate evaluation of bone health measurements.

## Background

Making weight and maintaining a competitive racing weight during the horse racing season is one of the major lifestyle challenges faced by jockeys [1]. The weight regulations of racing mean the potential pool of individuals deemed to naturally possess the physical characteristics to become a jockey appears to be reduced, and thus, there is increasing difficulty in achieving such a low body mass [2]. For jockeys, establishing a low body fat % (BF%) has become integral as they must align their body mass with the stipulated weight of the horse they ride in each race [3]. Furthermore, lean mass (LM) is identified as having significant interaction with bone mass variables such that the skeletal system adjusts and adapts in accordance with its physical environment via the mechanostat theory [4]. Thus, accurately quantifying fat mass (FM) and LM for jockeys is a priority as the effects of extremely low BF% and LM may increase the risk of injury and severe medical problems such as low bone mineral density (BMD) and cardiovascular complications [5, 6]. On the other hand, excess body fat can be perceived as ‘dead weight’ such that high levels of FM may have a negative impact on performance due to the demands of making weight [7]. Furthermore, low bone mass (preferred term for osteopenia [8]), osteoporosis and the risk of bony injuries are a serious concern for jockeys as previous research has shown a high prevalence of low BMD and impaired bone markers via a multitude of assessment methods [2, 9–13].

In the current literature on jockey body composition, measurements have been carried out to evaluate BF% using skinfold thickness and skinfold prediction equations and dual-energy X-ray absorptiometry (DXA), while bone status and bone characteristics have been determined using a variety of assessment techniques including DXA, bone turnover markers and peripheral quantitative computed tomography (pQCT). Furthermore, the classification criteria used for interpreting results have been inconsistent across the available literature [2, 9–12]. Thus, despite over a decade of body composition and bone health assessments in jockeys worldwide, the heterogeneity of the studies conducted makes comparisons, interpretation and prescription between the findings difficult. Therefore, standardised measurement techniques and accepted reference ranges in the racing industry could prevent unnecessary use of clinical resources and interventions. This narrative review aims to appraise the existing evidence on body composition and bone status in professional jockeys and to evaluate the assessment methods and classification criteria within the horse racing industry.

## Study Selection

A search strategy for this narrative review was conducted using electronic databases, including, PubMed, Google Scholar and Web of Science and consultation with experts in the field. Studies were identified using combinations of the following key search terms: ‘jockey’ AND ‘body composition’ OR ‘bone health’ OR ‘body fat’ OR ‘DXA’ OR ‘horse racing’ OR ‘bone mineral density’ OR ‘weight making’ OR ‘bone strength’. A manual search of reference lists of relevant studies was also reviewed to identify articles. The search was conducted up to and including 30 January 2021. Relevant articles published in English, available in full text and conducted in current and retired jockeys were assessed against the inclusion criteria described below.

### Inclusion Criteria

1. Body Composition: only studies that quantified surface anthropometry (skinfold thickness or estimated BF%) and or DXA (BF%, FM and or LM) outcome measurements.
2. Bone Health: only studies that quantified any of the following outcome measurements: BMD of whole-body, lumbar spine (LS), total hip, femoral neck, bone architecture (from pQCT), bone resorption and formation parameters (from metabolic biomarkers).

### Exclusion Criteria

1. Studies in languages other than English
2. Conference abstracts and case studies
3. Studies of amateur jockeys or other equestrian disciplines for example Show-jumping, Eventing or Dressage

In total, 23 body composition and 19 bone health jockey-specific studies were included in the narrative review.

## Body Composition of the Jockey

### Body Composition Assessment Methods

The two most commonly used methods for assessing body composition in jockeys have been skinfold thickness and DXA, with no other method of body composition assessment used in jockey research (Table 1). Both methods provide indirect measurement of body composition; however, DXA is a three-compartment model (FM, LM and bone mineral) compared to skinfold thickness which is a two-compartment model (FM and fat-free mass) [5]. The skinfold method is an accessible and practical means for monitoring changes in body fat of jockeys. The field-based technique is cost-effective, simple to use, time-efficient and has no limit to usage, allowing for repeated measurements throughout the racing season [5, 14]. The sum of skinfold thickness has a high degree of agreement with DXA FM measurement and thus can be used as a proxy for adiposity [14, 15]; however, body fat prediction equations including Durnin and Womersley [16], Jackson and Pollock [17] and Withers [18] are more frequently used to estimate a BF% in jockeys. Despite this, the accuracy of prediction equations is affected by a number of factors, including the cohort of athletes or type of sports included in the design of the equations, the skinfold callipers used, technicians’ expertise and the number and location of assessment sites used [19].

**Table 1:** Comparison of body composition and assessment methods in professional and retired jockeys^a^

Study (country)	Participants		Body composition							Assessment method
	Number (sex)	Age	Body mass (kg)	Height (cm)	BMI (kg m^−2^)	Body fat %	Lean mass (kg)	Fat mass (kg)	∑ of 7 (mm)	
Leydon and Wall [20] (New Zealand)	6 (m) and 14 (f) flat	23.5 ± 4.3 (m); 24.5 ± 6.7 (f)	52.8 ± 2.4 (m); 49.3 ± 3.4 (f)	162.3 ± 4.4 (m); 156.2 ± 4.0 (f)	20.1 ± 1.5 (m); 20.2 ± 1.5 (f)	11.7 ± 2.9 (m); 23.6 ± 3.8 (f)	–	–	–	DXA
Wilson et al. [12] (UK)	8 (m) and 8 (f) flat	26.0 ± 5.0 (m); 29.0 ± 8.0 (f)	57 ± 2.1 (m); 57.3 ± 3.5 (f)	167.0 ± 4.0 (m); 163.0 ± 5.0 (f)	–	12.5 ± 2.7 (m); 19.5 ± 2.5 (f)	45.7 ± 1.2 (m), 42.0 ± 3.3 (f)	–	–	DXA
Jackson et al. [13] (UK)	79 (m) and 37 (f) app; 69 (m) con	18.5 ± 1.9 (m) 19.3 ± 2.0 (f) app; 20.7 ± 2.0 con (m)	52.9 ± 2.9 (m) 51.6 ± 4.0 (f) app; 63.7 ± 3.6 con (m)	167.0 ± 6.0 (m) 157.0 ± 5.0 (f) app; 176.0 ± 5.0 con (m)	19.0 ± 1.4 (m) 20.8 ± 1.7 (f) app; 20.6 ± 1.3 con	14.6 ± 2.3 (m) 24.4 ± 3.7 (f) app; 15.7 ± 2.7 con	42.5 ± 2.6 (m), 36.4 ± 2.7 (f) app; 50.7 ± 3.1 con (m)	7.6 ± 1.3 (m), 12.4 ± 2.4 (f) app; 9.9 ± 1.7 con (m)	–	DXA
Wilson et al. [21] (UK)	32 (m) app	19.0 ± 1.8	56.0 ± 2.9	169.0 ± 4.7	–	–	45.1 ± 2.6	7.2 ± 1.8	–	DXA
Cullen et al. [22] (Ireland)	18 (m) tra; 8 (m) app	16.0 ± 1.0 tra; 18.0 ± 1.0 app	55.7 ± 5.5 tra; 54.9 ± 2.9 app	167.0 ± 5.0 tra; 169.0 ± 4.0 app	19.9 ± 1.7 tra; 19.2 ± 1.1 app	8.1 ± 1.7 tra 7.4 ± 1.3 app	–	–	45.7 ± 7.5 tra; 42.0 ± 8.0 app	Skinfold thickness Withers equation
Wilson et al. [23] (UK)	17 (m) app; 14 (m) sen flat	19.0 ± 2.0 app; 32.0 ± 7.0 sen	56.2 ± 2.0 app; 56.4 ± 3.0 sen	170.0 ± 5.0 app; 166.0 ± 5.0 sen	–	13.7 ± 2.6 app; 12.5 ± 1.9 sen	46.4 ± 2.0 app; 45.7 ± 3.1 sen	7.5 ± 1.7 app; 6.8 ± 1.4 sen	–	DXA
Labadarios et al. [24] (South Africa)	93 (m) flat and NH	27.8	52.9	160.9	–	11.0	–	–	–	Skinfold thickness
Warrington et al. [2] (Ireland)	17 (m) flat; 10 (m) NH	26.7 ± 7.6 flat; 28.3 ± 5.3 NH	53.1 ± 4.1 flat; 66.2 ± 2.9 NH	160.0 ± 10.0 flat; 173.0 ± 10.0 NH	19.9 ± 1.3 flat; 22.1 ± 0.8 NH	DXA: 9.0 ± 2.5 flat; 10.4 ± 4.0 NH SK: 7.9 ± 1.7 flat; 9.9 ± 1.6 NH	–	–	44.3 ± 10.2 flat; 56.1 ± 9.4 NH	DXA and Skinfolds Thickness Withers equation
Dolan et al. [1] (Ireland)	17 (m) flat; 10 (m) NH	26.7 ± 7.6 flat; 28.3 ± 5.3NH	53.1 ± 4.1 flat; 66.2 ± 2.9 NH	167.0 ± 10.0 flat; 173.0 ± 10.0 NH	19.9 ± 1.3 flat; 22.1 ± 0.8 NH	9 5 ± 2.5 flat; 10.4 ± 4.0 NH	49.7 ± 6.2 flat and NH	–	–	DXA
Hitchens et al. [25] (Australia)	7 (m) and 1 (f) app + sen	28.8 ± 10.1	55.1 ± 5.9	163.4 ± 7.1	20.6 ± 1.6	14.0 ± 3.5^b^	–	–	–	DXA
Dolan et al. [11] (Ireland)	20 (m) flat and NH	26.0 ± 3.0	61.1 ± 5.4	170.0 ± 7.0	21.4 ± 1.8	11.4 ± 5.6	52.5 ± 5.2	6.8 ± 3.6	–	DXA
Dolan et al. [26] (Ireland)	14 (m) flat; 16 (m) NH	25.0 ± 7.0 flat; 24.0 ± 4.0 NH	54.6 ± 3.6 flat; 64.3 ± 3.3 NH	165.0 ± 6.0 flat; 172.0 ± 5.0 NH	20.2 ± 1.6 flat; 21.9 ± 1.2 NH	8.3 ± 2.9 flat; 13.8 ± 6.0 NH	49.4 ± 3.8 flat; 53.7 ± 4.3 NH	4.4 ± 1.5 flat; 8.7 ± 3.9 NH	–	DXA
Wilson et al. [27]^c^ (UK)	9 (m) NH	24.0 ± 3.1	63.2 ± 4.7	172.0 ± 5.2	–	11.3 ± 2.2	51.6 ± 3.7	6.9 ± 1.7	–	DXA
Wilson et al. [3] (UK)	19 (m) flat; 18 (m) NH	27.0 ± 5.0 flat; 25.0 ± 5.0 NH	56.1 ± 2.9 flat; 65.3 ± 2.5 NH	167.0 ± 5.0 flat; 175.0 ± 5.0 NH	20.3 ± 1.4 flat; 21.4 ± 1.3 NH	13.0 ± 3.0 flat; 11.5 ± 3.3 NH	–	7.4 ± 1.0 flat; 8.2 ± 2.1 NH	–	DXA
Dolan et al. [28] (Ireland)	4 (m) flat; 5 (m) NH	24.0 ± 7.0 flat and NH	58.2 ± 5.3 flat and NH	168.0 ± 5.0 flat and NH	20.7 ± 1.7 flat and NH	9.0 ± 1.4 flat and NH	–	–	51.1 ± 8.0 flat and NH	Skinfold thickness Withers equation
Wilson et al. [29]^c^ (UK)	10 (m and f) flat and NH	32.0 ± 6.0	59.2 ± 4.6	167.0 ± 8.0	–	13.1 ± 5.9	47.1 ± 5.3	7.3 ± 3.5	–	DXA
O’Reilly et al. [30] (Hong Kong)	20 (m) flat	29.3 ± 7.8	53.8 ± 3.3	162.0 ± 6.0	20.5 ± 1.5	5.8 ± 2.6	50.8 ± 2.5^d^	3.2 ± 1.5^d^	42.9 ± 11.8	Skinfold thickness D & W equation
Poon et al. [9] (Hong Kong)	14 (m) flat	29.1 ± 6.1	52.8 ± 3.7	161.0 ± 5.0	20.3 ± 1.6	–	–	–	42.4 ± 9.1	Skinfold thickness J & P equation
Jeon et al. [31] (Korea)	10 (m) flat	31.8 ± 3.7	50.6 ± 1.9	157.5 ± 4.5	20.5 ± 1.4	14.4 ± 2.3	43.3 ± 1.7	7.3 ± 1.2	–	DXA
Dunne et al. [14] (Ireland)	35 (m) flat; 37 (m) NH	27.5 ± 9.6 flat; 27.5 ± 5.4 NH	56.1 ± 3.2 flat; 65.5 ± 3.4 NH	167.2 ± 5.5 flat; 175.6 ± 4.5 NH	–	15.0 flat; 14.6 NH	–	–	44.1 ± 10.2 flat^e^; 47.7 ± 10.5 NH^e^	DXA and skinfold thickness
Dunne et al. [32] (Ireland)	39 (m) flat; 46 (m) NH	26.8 ± 9.6 flat; 27.7 ± 5.9 NH	55.7 ± 3.2 flat; 65.7 ± 3.2 NH	167.3 ± 5.4 flat; 174.6 ± 4.4 NH	20.0 ± 1.3 flat; 21.6 ± 1.2 NH	14.9 ± 3.0 flat; 15.3 ± 3.4 NH	45.7 ± 3.1 flat; 53.4 ± 3.4 NH	8.0 ± 1.7 flat; 9.6 ± 2.3 NH	–	DXA
Cullen et al. [33] (Ireland)	Retired: 7 (m) flat; 12 (m) NH; 9 (m) dual	61.0 ± 6.0 flat; 59.0 ± 6.0 NH; 58.0 ± 7.0 dual	65.6 ± 11.0 flat; 77.9 ± 11.4 NH; 74.5 ± 7.0 dual	163.0 ± 5.0 flat; 167.0 ± 3.0 NH; 166.0 ± 3.0 dual	24.8 ± 4.0 flat; 28.1 ± 4.1 NH; 27.0 ± 2.7 dual	23.1 ± 6.8 flat; 27.1 ± 6.1 NH; 26.9 ± 7.3 dual	42.5 ± 5.3 flat; 49.5 ± 5.2 NH; 47.9 ± 5.2 dual	14.3 ± 7.0 flat; 20.7 ± 7.4 NH; 19.2 ± 6.2 dual	–	DXA
Mackinnon et al. [34] (UK)	Retired: 209 (m) all; 135 (m) 50+	56.1 ± 14.6 all; 64.7 ± 9.9 50+	–	–	24.5 ± 2.9 all; 25.0 ± 3.0 50+	–	–	–	–	Retired Athletes Questionnaire

Values presented as mean ± SD

*f* female, *m* male, *NH* national hunt, *app* apprentice, *con* conditional, *sen* senior, *tra* trainee, *SK* skinfold, *dual* flat and national hunt riding licence, *D & W* Durnin & Womersley, *J & P* Jackson and Pollock, *all* all jockeys recruited*, 50*+ jockeys ≥ 50 years of age

^a^Table is arranged based on sex and racing licence with studies of female jockeys first followed by male apprentice and conditional then senior and retired jockeys

^b^Based on 5 jockeys

^c^Baseline measurement of jockeys recruited for study intervention

^d^Estimated using skinfold prediction equations

^e^Sum of 8 skinfold thickness

In contrast, the DXA whole-body scan has been used to quantify BF%, FM and LM in jockeys. While the absolute values of FM and LM are preferred for assessing body composition by the International Olympic Committee [19], the accuracy of fat estimates in small lean athletes may not be reliable due to very low FM levels falling beyond the calibrated range of DXA [5]. Furthermore, the feasibility of DXA remains a barrier for some jockeys as well as the limited frequency it can be used due to the cumulative exposure to radiation resulting from diagnostic procedures [5, 35]. A standardised testing protocol should be used for both methods to reduce biological error and subsequently increase measurement reliability [5, 36]. Consistent pre-assessment preparation including overnight fasted and rested state, euhydrated and bladder void are recommended [37, 38]; however, due to the lifestyle and weight-sensitive nature of horse racing this can be challenging. Despite this, acute food fluid intake (< 500 g) prior to testing may only cause trivial effects to DXA measurements [39]. Furthermore, some studies have reported moderate dehydration [2, 21] and euhydration [12, 32] in jockeys prior to DXA scan, suggesting monitoring hydration status and providing regular reminders of the testing protocols is important to ensure accurate measurements particularly the assessment of LM [40].

### Anthropometric Measurements

The weight classifications for flat (start in stalls and compete over course distances of 1–4 km) and national hunt (NH) (start from a tape barrier and compete over course distances of 3.2–7.2 km that involve a number of hurdles) races vary from country to country with the allocated racing weights consisting of the jockey’s body mass, body protector, helmet and saddle [41]. Changes in minimum weight allocation between racing jurisdictions globally have historically been slow, with weight limits typically > 10% below the average reference population and not reflective of changes in mean body mass of the reference population in the past century [33, 42]. In Ireland and the UK, recent data of flat jockeys report an average weight of between 55.7 kg [32] to 56.2 kg [23]. Yet, compared to early research from Ireland, New Zealand and South Africa the body mass of flat jockeys has increased (average body mass: 52.9 [range 52.8 to 53.1 kg]) [2, 20, 24]. It is clear the height of flat jockeys has increased linearly in the same 30 year time period from 160.9 cm and 160.1 cm in South Africa [24] and Ireland [2], respectively, to 167.3 cm and 166.0 cm in Ireland [32] and the UK [23], respectively, while BMI has remained relatively unchanged. The increase in height may explain the rise in body mass of flat jockeys which may lead to the use of extreme weight loss techniques, including severe energy restriction and excessive dehydration when attempting to make the weight; however, further research is warranted to investigate flat jockeys’ body mass. In contrast, the weight and height variation in NH jockeys is less evident over the last decade. Body mass and height have remained constant at between 64.3 to 66.2 kg and 172.0 to 175.0 cm [2, 3, 26, 32].

Newly licensed flat and NH jockeys are termed apprentice and conditional, respectively, and while they race under the same rules as professionals (also referred to as seniors), they are given a reduction in weight (‘claiming allowance’; 0.5–1.4 kg, depending upon number of races won) to compensate for their race riding inexperience and encourage trainers to allocate them more riding opportunities [41]. However, this weight advantage places greater emphasis on young jockeys to ride at significantly reduced weights [41]. The reported weights of apprentice jockeys vary. In Ireland, Cullen et al. [22] recorded an average body mass of 54.9 kg, yet in the UK, the body mass of apprentice jockeys averaged as low as 52.9 kg [13] up to 56.0 kg [21, 23]. Conversely, the height of apprentice jockeys is consistent in the literature (average height ranged between 167.0 and 170.0 cm) and thus indicates the potential challenges of making weight for young aspiring jockeys [13, 21–23]. While data on conditional jockeys are limited, the recorded body mass (63.7 kg) and height (176.0 cm) would suggest that the average stature is increasing and consequently making weight will become a challenge for these jockeys as they progress throughout their career [13]. Similarly, there is a lack of literature on female jockeys despite competing under same competition rules as male jockeys, yet the available data for female flat jockeys show that they are shorter than their male counterparts (average height ranged between 156.2 and 163.0 cm) [12, 13, 20]. Their shorter stature may allow female jockeys to achieve the allocated racing weight without the need of using acute weight loss strategies like fasting and severe dehydration [13].

### Surface Anthropometry

Studies on jockeys from Ireland and Hong Kong have recorded absolute skinfold thickness in millimetres (mm). Warrington et al. [2] found a significant difference between professional flat and NH jockeys using a sum of 7 measurement (minus iliac crest skinfold site) (44.3 mm and 56.1 mm, flat and NH, respectively). In contrast, Dunne et al. [14] reported no significant difference in a large-scale study of skinfold thickness between professional jockeys using a sum of 8 measurement (inclusion of iliac crest skinfold site) (44.1 mm and 47.7 mm, flat and NH, respectively). Despite the inclusion of an additional skinfold site, flat jockeys in both studies presented with comparable absolute values. Data in both studies was collected by an International Society for the Advancement of Kinanthropometry (ISAK) accredited anthropometrist. Notwithstanding the possibilities of inter-investigator variation, the similarities between the two flat jockey cohorts may be due to a total reduction in subcutaneous fat adiposity in the group assessed using the sum of 8 measurement as reflected by the lower skinfold values recorded in NH jockeys. Similar recordings of absolute skinfold thickness using a sum of 7 measurement have been reported in professional flat jockeys from Hong Kong (42.9 mm and 42.4 mm, [30] and [9], respectively). These findings in professional jockeys are consistent with Irish apprentice jockeys (42.0 mm) [22]. Taken together skinfold thickness in absolute terms can provide an accurate indication of adiposity when monitoring body fat of individual jockeys and eliminate the error associated with converting skinfolds into BF% [19]. However, to ensure consistency with assessment and reporting of results, a standardised protocol for skinfold measurement using approved measurement procedures by ISAK is recommended for national and international comparisons [19].

The two most commonly used skinfold prediction equations for estimating BF% in jockeys are the Withers [18] and Durnin and Womersley [16] equations. When assessing body composition in a group of Irish jockeys using the Withers equation, estimated BF% was reported as 7.4% for apprentice jockeys [22], 7.9% for flat and 9.9% for NH jockeys [2] and between 9.0 and 8.9% in a group of flat and NH jockeys undertaking an acute weight loss trial [28]. In contrast, O’Reilly et al. [30] reported a lower BF% (5.8%) in a group of flat jockeys from Asia, including 9 Caucasian jockeys using the Durnin and Womersley equation. Conversely, the BF% was higher (11.0%) in a large group (*n* = 93) of South African jockeys using the Durnin and Womersley equation [24]. The differences between the studies may result from the technical error between technicians and the expected variation in age and sample size between different cohorts. Despite this, prediction equations including Withers and Durnin and Womersley can introduce additional variability due to differences in the sites selected for skinfold assessment and the sample populations (sport-specific vs. non-athlete level) used in the design of the equations [43, 44].

Dunne et al. [14] investigated the accuracy and variability of prediction equations at estimating BF% in a group of 72 professional flat and NH jockeys relative to DXA. The authors found that commonly used prediction equations, Withers (6.9%), Durnin and Womersley (7.1%), Evans (8.0%) [45] and Reilly (9.2%) [46] display considerable variability in the assessment of BF% and underestimate BF% in comparison to DXA (14.8%). Despite athlete-specific equations such as Withers and Evans that have included subjects from multidisciplinary and multi-ethnic backgrounds, regional differences in fat distribution between sample populations may inherently lack precision when applied to a specific sporting population like jockeys. Thus, caution must be taken when providing jockeys feedback or making comparisons with data obtained from equation estimates. Consequently, Dunne et al. [14] developed jockey-specific equations for the assessment of BF% in flat and NH jockeys. The novel prediction equations showed good predictive power (84% and 83%, flat and NH, respectively) and a high level of agreement with DXA using the Bland Altman Plots method. The jockey-specific equations, therefore, provide an accurate field-based method to estimate BF% relative to DXA in flat and NH jockeys. Despite this, validation of the equations is required in a separate jockey population in order to test the validity of the novel equations. For practitioners working in the horse racing industry, the jockey-specific equations provide a practical and non-invasive method to assess and track changes in BF% for the health and performance of jockeys [14].

### Whole-Body Assessment

The DXA-derived BF% has also been reported to be equally variable across jockey cohorts, including flat and NH jockeys in Ireland and the UK. In Ireland, Warrington et al. [2] and Dolan et al. [26], despite reporting similar BF% for flat jockeys (9.0% vs. 8.3%), showed a large difference between NH jockeys (10.4% vs. 13.8%). In the UK, Wilson et al. [3] recorded a higher BF% in flat (13.0%) compared to NH (11.5%) jockeys. More recently, Irish jockeys have presented with higher BF% for flat (14.9%) and NH (15.3%) riders [32]. The higher levels of BF% may be related to the broader age profile and larger sample size in recent research or variance in jockey conditioning and dietary intake. Furthermore, the GE Lunar Prodigy has been used for the data collection in Irish jockeys, while the Hologic model was used to assess jockeys in the UK. The use of different DXA models and software may introduce additional variation when making comparisons, including different beam technology, scan speed and algorithms [37].

Studies reporting the BF% of female jockeys show a variation based on age profile. Research by Jackson et al. [13] and Leydon and Wall [20], found young female flat jockeys (19.3 vs. 23.5 years of age) presented with an average BF% of 24.4% and 23.6%, respectively. In contrast, Wilson et al. [12] recorded 19.5% BF% in a group of female jockeys with an average age of 29.8 years. Due to the dearth of current data available, further research is required to explore the body composition of female jockeys. Apprentice and conditional jockeys appear to display comparable levels of BF% to senior jockeys. Jackson et al. [13] reported a lower BF% in apprentice (14.6%) compared to conditional (15.7%) jockeys, noting that the DXA scans were performed on three different scanning models. Despite this, results suggest the BF% of young newly licensed jockeys is similar to senior jockeys (flat 14.9% vs. NH 15.3%) [32]. Notwithstanding this, Wilson et al. [23] found higher BF% in a group of apprentice jockeys (13.7%) compared to professional flat jockeys (12.5%). It is worth highlighting the potential measurement error when assessing FM in small lean athletes [5, 47]. Therefore, to minimise error when assessing BF% a standardised protocol using the same model is recommended [19].

Absolute FM, as might be expected, was similar in variation to the BF% with flat (4.4 kg) jockeys having lower FM than NH (8.7 kg) jockeys [26]. However, a less noticeable difference has been reported between flat (7.4 kg) and NH (8.2 kg) jockeys [3], and this is comparable to a recent publication by Dunne et al. [32]. The evolving evidence suggests a trend towards higher FM values in professional jockeys (8.0 kg vs. 9.6 kg, flat and NH, respectively) [32]. Higher levels of absolute FM may have a positive influence on BMD [48]; however, further research is necessary to explore this relationship in jockeys. In apprentice male jockeys, the FM values remain consistent as studies by Jackson et al. [13] and Wilson et al. [21, 23] have reported absolute FM between 7.2 and 7.6 kg. A retrospective study of body composition analysis by Wilson et al. [21] reported FM values ranging from 3.7 to 10.4 kg (mean 7.2 kg) in apprentice jockeys. The authors suggested that despite the low levels of FM, apprentice jockeys continue to struggle with making minimum weight and subsequently risk muscle catabolism to achieve the assigned riding weights [21]. In female apprentice jockeys, the FM values (12.4 kg) were higher than their male counterparts (7.6 kg) [13]. While the higher values of FM in female jockeys are indicative of the reproductive system, no clear guidelines exist for optimum FM in female or male jockeys due to the variability between individuals and the inherent errors associated with body fat assessment [6]. For instance, female jockeys have lower FM values than other female athletes in weight category combat sports, including boxing, judo and wrestling [49].

In Irish jockeys, LM values were found to be lower in flat (49.4 kg) and NH (53.7 kg) jockeys compared to boxers (58.1 kg) and recreationally active controls (58.0 kg) [26]. The authors suggested the proportionality between bone and LM may help identify the low bone mass [11]. Similar LM values were reported for NH (53.4 kg) jockeys but not flat jockeys (45.7 kg) [32]. Flat jockeys may have greater difficulty at achieving and maintaining LM due to weight-making practices that use severe dehydration (sauna use, hot baths and sweat suits) and excessive cardio-based exercise (48%) as a means of rapid weight loss [50]. These findings are consistent in male flat jockeys (45.7 kg) from the UK [12, 23]. When LM is expressed relative to height (lean mass index (LMI)), flat (18.2 kg m^−2^) and NH (18.3 kg m^−2^) jockeys possess less LM compared to boxers (19.2 kg m^−2^); however, there is no difference compared to the active controls (18.7 kg m^−2^) [26]. Differences in training load, namely multi-directional speed and power exercise, and the reduced frequency of chronic weight loss may favour greater LM development and maintenance in the boxing group [26]. Dunne et al. [32] reported lower LMI levels for flat (16.4 kg m^−2^) and NH (17.5 kg m^−2^) jockeys in Ireland. This may be due to the lower LM values in flat and slightly increased stature in NH jockeys compared to Dolan et al. [26]. The LM values in male apprentice jockeys differ between studies in the UK as Wilson et al. [21, 23] observed higher LM values (45.1 kg and 46.4 kg, [21] and [23], respectively) compared to Jackson et al. [13] (42.5 kg). While the average age (19.2 years) was similar in the reported studies, the differences in LM values of male flat jockeys’ in the UK may be due to recruitment bias. For example, participants in smaller-scale studies may possibly be actively interested in body composition and achieving an optimum LM value. Nonetheless, the physical maturity of young jockeys can differ significantly between study cohorts, and lower LMI values emphasise this in apprentice (15.3 kg m^−2^) and conditional (16.4 kg m^−2^) jockeys [13]. Furthermore, DXA assessment of LM in jockeys requires careful pre-scan presentation as results are affected by acute changes in hydration and glycogen status [40, 51]. Thus, where strict measurement protocols are impractical caution is advised when interpreting results as lifestyle factors frequently experienced by jockeys such as rapid weight loss using food and fluid restriction could increase the variability of accurate LM results [40, 51].

The long-term body composition fluctuations of jockeys are less well known. Cullen et al. [33] is the only available study to report the BF%, FM and LM values of retired jockeys. Noticeably, FM was higher in retired flat (14.3 kg) and NH (20.7 kg) jockeys than professional jockeys in the present day yet BF% was comparable to Irish males ≥ 50 years (28.1%) [52]. This may be due to a phenomenon known as fat overshooting, an increase in FM following recovery from weight cycling. Consequently, a career of chronic weight cycling to make weight for racing may result in retired jockeys becoming predisposed to obesity [53]. However, ranges for body mass index were below the classification for diagnosing obesity (30 kg m^−2^), and despite the participants reporting difficulty with attaining stipulated racing weights, it was acknowledged that the challenges of making weight and chronic weight cycling were less extreme compared to present-day jockeys [33]. The authors highlighted a 47% increase in mean body mass (37.0 to 54.5 kg) of trainee jockeys entering the Racing Academy and Centre of Education between 1978 and 2012, yet there has been a disproportionate (10%) increase in weight allocation (47.7 to 52.7 kg) for apprentice jockeys in the same time period. Furthermore, the retired jockeys had a shorter stature compared to present-day jockeys, and therefore, making weight may have been less challenging [33]. In contrast, Mackinnon et al. [34] reported 42% of retired jockeys over 50 years of age were obese. While the study used self-reported height and weight measurements, further research is warranted to track the long-term impact on body composition of chronic weight cycling in retired jockeys.

## Bone Health of the Jockey

### Bone Assessment Methods

Several measurement techniques have been used to assess jockeys’ bone health, including DXA, pQCT, bone turnover markers and questionnaires (Table 2). DXA is the most popular method for assessing the bone health of jockeys. The two-dimensional assessment can be used to detect low BMD and is recognised by the World Health Organization as the reference method of diagnosing osteoporosis (International Society for Clinical Densitometry (ISCD), [8]). Yet, the ISCD state that BMD alone cannot be used to diagnose osteoporosis in men under age 50. Moreover, a *Z*-score is preferred when reporting BMD in males younger than age 50 and in females prior to menopause [8]. However, *T*-scores were reported in early literature of jockey BMD making direct comparisons to studies presenting Z-scores difficult. Furthermore, comparisons between absolute values of BMD, the only consistent measurement between studies, are problematic due to different DXA models and advances in software, variation of measured and reported BMD sites across the jockey literature, namely LS (L2–L4 vs. L1–L4) and whole-body only assessments. While DXA technology can provide measurements of bone length and width, estimates of bone depth are required to reflect bone volume. This is important when interpreting the BMD of individuals of varying size or where bone size may change, such as growing adolescents [54]. For example, an increase in BMD may result from the greater bone size and not an increase in volumetric BMD. Hence, a volumetric adjustment to approximate the effects of bone depth and body size (bone mineral apparent density (BMAD) has been developed for DXA.

**Table 2:** Comparison of bone parameter measurements and results in studies of male and female professional and retired jockeys^a^

Study (country)	Participants		Assessment method	Bone parameters measured	Results	
	Number (sex)	Age				
Greene et al. [10] (Australia)	11 (m) and 14 (f) app	20.1 ± 4.9	pQCT	Tibial and radial bone variables: trabecular area and density, BMC, TBA, cortical area and density, SSI	Tibia: cortical area (mm^2^)—66% proximal: 311.4 ± 40.5 Cortical density (g cm^2^)—66% proximal: 1163.6 ± 38.1 SSI (mm^3^) 4% distal 1683.3 ± 123.7; 66% proximal 1953.9 ± 326.8 Radius: cortical area (mm^2^)—66% proximal 81.4 ± 16.7 SSI (mm^3^) 4% distal 391.9 ± 69.4; 66% proximal 297.3 ± 88.3	
Silk et al. [55]^b^ (Australia)	17 (m) app	22.3 ± 5.0 (supp) versus 19.3 ± 1.8 (pla)	pQCT and bone markers	4% distal tibia: trabecular area, content and density, TD, TBA, bone strength index 66% proximal tibia: cortical content, area, density, thickness, TBA SSI tibia, P1NP, CTX	Trabecular density (mg cm^2^): 241.0 ± 28.1 (supp); 246.8 ± 33.9 (pla) Trabecular area (mm^2^): 861.1 ± 160.2 (supp); 854.7 ± 126.7 (pla) Cortical density (mg cm^2^): 1101.5 ± 24.7 (supp); 1113.4 ± 22.1 (pla) Cortical area (mm^2^): 267.1 ± 29.3 (supp); 263.4 ± 43.8 (pla) SSI tibia (mm^3^): 2100.5 ± 329.2 (supp); 2140.0 ± 489.6 (pla)	CTX (ng/L): 371.3 ± 201.0 (supp); 380.0 ± 141.1 (pla) P1NP (μg/L): 104.2 ± 46.4 (supp); 108.9 ± 31.6 (pla)
Waldron-Lynch et al. [56] (Ireland)	9 (m) flat; 3 (m) NH	25.5 ± 5.0	Bone markers	NTx, fDPD, P1NP, bone ALP, OC		NTx (nmol/mmol)^c^: 99.4 ± 72.6 fDPD (nmol/mmol)^c^: 6.98 ± 3.27 P1NP (μg/L): 118.8 ± 76.1 Bone ALP (μg/L): 18.8 ± 10.9 OC (μg/L): 20.8 ± 11.3
Dolan et al. [11] (Ireland)	20 (m)	25.9 ± 3.3	DXA and bone markers	BMD: WB WB, LS (L2–4), FN BMAD: LS (L2–4), FN NTx, P1NP	BMD (g/cm^2^) WB: 1.134 ± 0.05 LS: 1.11 ± 0.08 FN: 1.06 ± 0.09 BMAD (g/cm^3^) LS: 0.137 ± 0.01 FN BMAD: 0.39 ± 0.04	NTx (nmol L^−1^): − 0.35 ± 0.88 P1NP (ng mL^−1^): 88.62 ± 46.69
Wilson et al. [3] (UK)	19 (m) flat; 18 (m) NH	27.0 ± 5.0 flat; 25.0 ± 5.0 NH	DXA and bone markers	BMD *T*-score: WB CTx	*T*-score WB: − 0.87 ± 0.77 flat; − 0.11 ± 1.00 NH	CTx (μg L^−1^): 0.46 ± 0.21 flat; 0.36 ± 0.14 NH
Jackson et al. [13] (UK)	79 (m) and 37 (f) app; 69 (m) con	18.5 ± 1.9 (m) 19.3 ± 2.0 (f) app; 20.7 ± 2.0 (m) con	DXA	BMD and BMC: LS (L1–L4), FN, TH BMAD: LS (L1–L4), FN *Z*-score ≤ − 1.0: LS (L1–L4), FN, TH *Z*-score ≤ − 2.0: LS (L1–L4), FN	BMD (g/cm^2^) LS: 0.876 ± 0.091 (m); 0.987 ± 0.083 (f) app; 0.969 ± 0.087 con FN: 0.831 ± 0.070 (m); 0.840 ± 0.077 (f) app; 0.935 ± 0.093 con TH: 0.924 ± 0.084 (m) 0.959 ± 0.075 (f) app; 1.017 ± 0.100 con BMAD (g/cm^3^) LS: 0.114 ± 0.011 (m); 0.132 ± 0.013 (f) app; 0.118 ± 0.011 con FN: 0.155 ± 0.113 (m); 0.176 ± 0.023 (f) app; 0.168 ± 0.023 con *Z*-score ≤ − 1.0 *n* [%] LS: 60 [76.0] (m); 6 [16.2] (f) app; 36 [52.2] con FN: 27 [34.2] (m); 3 [8.1] (f) app; 6 [8.7] con TH: 33 [41.8] (m); 1 [2.7] (f) app; 7 [10.1] con *Z*-score ≤ − 2.0 *n* [%] LS: 23 [29.1] (m); 1 [2.7] (f) app; 9 [13.0] con FN: 1 [1.3] (m) app	
Leydon and Wall [20] (New Zealand)	2 (m) and 9 (f) app; 4 (m) and 5 (f) sen	20.5 ± 3.8 app; 28.7 ± 5.0 sen	DXA	BMD *T*-scores: WB, LS, TH	*T*-scores: WB: − 1.80 ± 0.76 LS: − 0.36 ± 1.0 TH: − 0.54 ± 1.1	
Wilson et al. [12] (UK)	8 (m) and 8 (f) flat	25.0 ± 5.0 (m); 29.0 ± 8.0 (f)	DXA	BMD: LS, TH BMD *Z*-score: LS, TH	BMD (g/cm^2^) LS: 0.90 ± 0.14 (m); 1.02 ± 0.13 (f) TH: 0.89 ± 0.1 (m); 0.87 ± 0.15 (f) *Z*-score LS: − 1.6 ± 1.3 (m); − 0.3 ± 0.8 (f) TH: − 1.2 ± 1.0 (m); − 0.02 ± 0.8 (f)	
Wilson et al. [23] (UK)	17 (m) app; 14(m) sen flat	19.0 ± 2.0 app; 32.0 ± 7.0 sen		*Z*-score: LS, TH	*Z*-score LS: − 1.3 ± 1.4 app; − 1.5 ± 1.0 sen TH: − 0.9 ± 1.1 app; − 0.8 ± 0.7 sen	
Hitchens et al. [25] (Australia)	5 (m and f) app and sen	28.8 ± 10.1	DXA	BMD: WB	BMD (g/cm^2^) WB: 1.157 ± 0.07	
Warrington et al. [2] (Ireland)	17 (m) flat; 10 (m) NH	26.7 ± 7.6 flat; 28.3 ± 5.3 NH	DXA	BMD: WB, LS, TH BMD *T*-score: WB, LS, TH	BMD (g/cm^2^) WB: 1.05 ± 0.07 flat; 1.21 ± 0.06 NH LS: 1.12 ± 0.11 flat; 1.22 ± 0.15 NH TH: 0.99 ± 0.1 flat; 1.08 ± 0.13 NH *T*-score WB: − 1.23 ± 0.99 flat; − 0.16 ± 0.82 NH LS: − 0.91 ± 0.84 flat; − 0.3 ± 1.18 NH TH: − 0.67 ± 0.87 flat; − 0.11 ± 1.01 NH	
Dolan et al. [26] (Ireland)	14 (m) flat; 16 (m) NH	25.0 ± 7.0 flat; 24.0 ± 4.0 NH	DXA	BMD, BMC and BA: WB, LS (L2–4), FN BMAD: LS (L2–4), FN	BMD (g/cm^2^) WB: 1.09 ± 0.6 flat; 1.17 ± 0.05 NH LS: 1.10 ± 0.09 flat; 1.15 ± 0.1 NH FN: 1.05 ± 0.07 flat; 1.07 ± 0.11 NH BMAD (g/cm^3^) LS: 0.14 0.01 flat; 0.14 0.01 NH FN: 0.39 ± 0.04 flat; 0.38 ± 0.05 NH	
Wilson et al. [29]^b^ (UK)	10 (m and f) flat and NH	32.0 ± 6.0	DXA	*Z*-score: LS, TH	*Z*-score LS: − 1.32 ± 0.76 (Pre) TH: − 1.04 ± 1.2 (Pre)	
O’Reilly et al. [30] (Hong Kong)	20 (m) flat	29.3 ± 7.8	DXA	BMD: forearm, calcaneus *T* and *Z*-score: forearm, calcaneus	BMD (g/cm^2^) Forearm: 0.57 ± 0.07 (left); 0.56 ± 0.06 (right) Calcaneus: 0.46 ± 0.12 (left); 0.51 ± 0.06 (right) *Z*-score Forearm: 0.02 ± 0.99 (left); − 0.16 ± 1.15 (right) Calcaneus: − 1.43 ± 0.77 (left); − 1.22 ± 0.79 (right)	
Poon et al. [9] (Hong Kong)	14 (m) flat	29.1 ± 6.1	DXA	BMD: calcaneus, forearm *T* and *Z*-score: calcaneus, forearm	BMD (g/cm^2^) Calcaneus: 0.50 ± 0.06 (left); 0.51 ± 0.07 (right) Forearm: 0.58 ± 0.08 (left); 0.56 ± 0.06 (right) *Z*-score Calcaneus: − 1.60 ± 0.90 (left); − 1.42 ± 0.94 (right) Forearm: − 0.01 ± 1.06 (left); − 0.22 ± 0.92 (right)	
Jeon et al. [31] (Korea)	10 (m) flat	31.8 ± 3.7	DXA	BMD: WB BMC *T* and *Z*-score: WB	BMD (g/cm^2^) WB: 1.155 ± 0.126 BMC *Z*-score WB: 0.00 ± 1.35	
Dunne et al. [32] (Ireland)	39 (m) flat; 46 (m) NH	27.7 ± 5.9 NH; 26.8 ± 9.6 flat	DXA	BMD and BMC: WB, LS (L1–4), FN, TH BMAD: LS (L1–4), FN *Z*-score ≤ − 1.0: LS (L1–4), FN, TH *Z*-score ≤ − 2.0: LS (L1–4), FN	BMD (g/cm^2^) WB: 1.115 ± 0.081 flat; 1.244 ± 0.075 NH LS: 1.072 ± 0.113 flat; 1.163 ± 0.109 NH FN: 1.026 ± 0.115 flat; 1.100 ± 0.118 NH TH: 0.985 ± 0.106 flat; 1.057 ± 0.111 NH BMAD (g/cm^3^) LS: 0.125 ± 0.014 flat; 0.124 ± 0.012 NH FN: 0.389 ± 0.048 flat; 0.395 ± 0.044 NH *Z*-score ≤ − 1.0 *n* [%] LS: 23 [59] flat; 14 [30] NH FN: 10 [26] flat; 3 [7] NH TH: 19 [49] flat; 6 [13] NH *Z*-score ≤ − 2.0 *n* [%] LS: 8 [21] flat; 3 [7] NH TH: 2 [5] flat; 1 [2] NH	
Cullen et al. [33] (Ireland)	28 (m) flat and NH retired	59.0 ± 6.0	DXA and bone markers	BMD: WB, LS, PF *T*-score: WB, LS, PF P1NP, CTx	BMD (g/cm^2^) WB: 1.113 ± 0.142 LS: 1.008 ± 0.163 PF: 0.989 ± 0.154 *T*-score WB: − 1.1 ± 0.8 LS: − 0.7 ± 1.1 PF: − 0.8 ± 0.9	CTx: 0.395 ± 0.207 P1NP: 39.66 ± 14.43
Mackinnon et al. [34] (UK)	135 (m) retired 50+	64.7 ± 9.9	Questionnaire	Self-reported osteoporosis	7.4% of the retired jockeys reported as having osteoporosis compared to 1.6% of the reference population	

Values are mean ± SD, *f* female, *m* male, *NH* national hunt, *app* apprentice, *con* conditional, *sen* senior, *tra* trainee, *WB* whole body, *LS* lumbar spine, *TH* total hip, *PF* proximal femur, *FN* femoral neck, *BMC* bone mineral content, *BMD* bone mineral density, *BMAD* bone mineral apparent density, *CTx* C-terminal telopeptide of type I collagen, *P1NP* procollagen type 1 amino terminal propeptide, *Bone ALP* bone alkaline phosphate, *OC* intact osteocalcin, *NTx-1* N-telopeptides of type 1 collagen, *pQCT* peripheral Quantitative Computed Tomography, *TBA* total bone area, *fDPD* free deoxypyridinoline cross-links*, Supp* supplement group*, Pla* placebo group, *RCT* randomised controlled trial, *SSI* strain strength index, *50*+ jockeys ≥ 50 years of age

^a^Table is arranged based on assessment method and racing licence with studies of pQCT first followed by bone turnover markers and DXA in present-day jockeys then retired jockeys

^b^Baseline measurement of jockeys recruited for study intervention

^c^Expressed as a ratio with urine creatinine concentration

In contrast, pQCT offers a three-dimensional evaluation of bone volume and provides the ability to distinguish between cortical and trabecular bone [57]. For jockeys, this assessment can evaluate the bone size, bone geometry and a surrogate marker of bone strength using a strain strength index (SSI). Despite this, the pQCT has several drawbacks, including cost, limited to assessing only peripheral sites, relatively high dose of radiation and higher precision error compared to DXA [58]. Measurement of blood bone markers (resorption markers: C-terminal telopeptide of type I collagen (CTx); formation markers: bone alkaline phosphatase (bone ALP), procollagen type 1 N-propeptide (P1NP), intact osteocalcin (OC) and urine (resorption markers: N-telopeptides of type 1 collagen (NTx), free deoxypyridinoline cross-links (fDPD)) can help detect the activity of bone metabolism and provide complementary information to bone density and bone quality assessments in jockeys [59]. However, pre-analytic factors and measurement techniques associated with sample collection can lead to inaccuracies. For instance the timing, posture, fasting status, phase of menstrual cycle and hydration status are all factors that can alter the quality of blood and urine samples [60]. Jockeys typically start work (riding out) early in the morning (6.30–7.30 a.m.); thus, markers that are dependent on time of collection due to circadian rhythm such as CTx (must be after an overnight fast) can cause logistical issues [59]. Moreover, the acute and chronic effects of diet and exercise may also impact the quality of blood and urine samples for analysis of bone turnover markers [36, 59]. Nonetheless, serum CTx and P1NP are recognised by the International Osteoporosis Foundation (IOF) and the International Federation of Clinical Chemistry and Laboratory Medicine (IFCC) as the reference markers for bone resorption and bone formation, respectively [59]. Therefore, to ensure consistency and comparability of data, standardisation of measurement is critical, including reporting the timing of collection, use of immunoassays and reagents, and exercise activity [59].

### Bone Geometry and Structure

Using a pQCT scanner, Greene et al. [10] found that apprentice jockeys had reduced cortical cross-sectional area (bone size, shape and density) at the distal tibia and radius sites than age and sex-matched controls. However, the authors found greater trabecular density at the ultra-distal radius and SSI at the radial mid-shaft compared to controls. This unexpected outcome suggests site-specific habitual loading of riding racehorses may have positively influenced bone strength at the forearms. This would suggest that additional loading at site-specific locations may offer an alternative mechanism through which jockeys could build stronger bones [10]. In a randomised controlled trial, Silk et al. [55] investigated the bone properties via pQCT and bone markers of young male jockeys before a 6-month calcium and vitamin D supplementation trial. The study reported a higher SSI at the tibia compared to Greene et al. [10]. Despite the similar age profile of the study cohorts, Greene et al. [10] recruited male and female jockeys and this may have resulted in a lower average SSI [61]. Moreover, a high dropout rate was reported following the clinical trial leading to a reduced sample size for baseline analysis [55]. This might suggest that the participants that completed the study may have been actively interested in optimising their health and well-being.

### Bone Turnover Markers

The bone turnover markers for bone metabolism in Irish jockeys have been explored [11, 56], with evidence indicating abnormal bone turnover in a combined group of flat and NH jockeys compared to young, healthy individuals. Markers of resorption (NTx) were elevated compared to bone formation (P1NP) leading to increased bone loss. The high bone turnover is likely the result of low intakes of calcium combined with energy deficiency and chronic weight cycling [56]. Wilson et al. [3] and Silk et al. [55] reported similar findings of higher than the expected bone resorption (CTx) in a group of flat and NH, and apprentice jockeys, respectively. Yet, the average CTx values were within the reference range for both groups. Furthermore, no significant difference was reported between the jockey groups [3]. The contrast in results to those of Dolan et al. [11] and Waldron-Lynch et al. [56] may be due to the analyses of different bone resorption markers. In retired jockeys, Cullen et al. [33] reported bone resorption (CTx) and bone formation (P1NP) markers to be within the normal reference ranges. The use of non-jockey control groups and clinical norms for interpreting the markers of bone turnover in jockeys may be misleading, as their comparison may not provide accurate reference values due to the atypical lifestyle of jockeys [3, 55]. Thus, the accumulation of further bone turnover data using the markers of reference standard (P1NP and CTx) is necessary to establish jockey-specific reference ranges and subsequently, provide an appropriate comparison for bone turnover [60].

### Bone Density

Research of bone density in jockeys worldwide has reported low BMD and a high incidence of whole-body, total hip and LS low bone mass in professional jockeys [2, 12, 13, 20, 26, 30]. In Ireland, Warrington et al. [2] using BMD *T*-scores reported whole-body, LS (L2–L4) and total hip low bone mass in 59% of flat and 40% of NH jockeys. The lower weight ranges for flat jockeys could be a possible causative factor for poor BMD values, such that lower body mass as a result of restrictive dieting and extreme weight-making practices may lead to reduced BMD [2]. Wilson et al. [3] observed similar results in the UK as flat jockeys had significantly lower whole-body BMD *T*-scores than NH jockeys; however, no other bone sites were assessed. A comparative study investigating the difference between jockeys and other weight category athletes found similar results as flat and NH jockeys had reduced BMD at the whole-body, LS (L2–L4) and femoral neck compared to two separate groups of physically active controls and boxers [26]. The authors did not include a *Z* or *T*-score, yet it was suggested the difference in BMD between jockeys and boxers could be identified independently of LM and height. In other words, the development of whole-body BMD in the boxing group is consistent with the assertion that high impact activity provides osteogenic benefits that prevail over the benefits associated with LM alone [26]. Therefore, participating in high impact sports may convey a protective effect on BMD, which may overcome the negative osteogenic effects of rapidly reducing body mass [26]. The protective effect of high impact activity does not appear to be afforded to jockeys, with horse riding gait (walk, trot and canter), producing low impact levels at the lower limbs similar to walking on the ground [62]. Moreover, many refrain from weight-bearing exercise because of the commonly held yet unfounded belief that exercise will increase LM [41].

In Hong Kong, Poon et al. [9] and O’Reilly et al. [30] reported a high prevalence of low bone mass in flat jockeys, yet a comparison of BMD values to other studies is not possible as the model of DXA scanner (OsteoSys EXA-3000) was limited to measuring BMD at the forearms and calcaneus only. Similarly, Jeon et al. [60] observed low BMD in a group of Korean flat jockeys; however, the presented results suggest bone mineral content was used to calculate *Z* and *T*-scores and subsequently classify two jockeys with low bone mass and one with osteoporosis. Furthermore, the results are likely to be biased as the cohort chosen to complete the DXA scan were selected based on their chronic (> 5 years) use of extreme weight loss practices. Recently, Dunne et al. [32] reported low BMD *Z*-scores at the LS (L1–L4) (44%), femoral neck (15%) and total hip (29%) in Irish jockeys. Consistent with previous studies, flat jockeys had significantly lower BMD at all measured sites than NH jockeys. Additional analysis of physical and lifestyle factors associated with bone density in jockeys found that the practice of weight cutting and timing of weight cut were negatively associated with bone markers in flat and NH jockeys, respectively. The authors suggested the repeated cycle of weight-making over a short time frame may lead to immediate and longer-term health risks in jockeys [32]. Despite this, further research is required to substantiate these claims as estimates of bone density from general population data may be misleading [63]; thus, a jockey-specific reference database for *Z*-scores should be considered for future comparisons. Moreover, there remains a lack of conclusive evidence demonstrating the harms of extreme chronic weight cycling, suggesting the need for future studies to explore the various health outcomes, including acute injury risk and chronic morbidity [42].

One study investigating apprentice and conditional jockeys indicated that 76% of male apprentice and 52% of male conditional jockeys have low LS (L1–L4) BMD with 29% and 13%, respectively, reporting *Z*-scores <  − 2.0, a score that is below the expected range for age [13]. This large-scale assessment conducted over two and a half years measured BMD at the LS, total hip and femoral neck of newly licensed male and female jockeys (*n* = 186) in the UK. Low BMD values in male jockeys appear to stay constant throughout their racing career as Wilson et al. [23] showed no significant difference in either LS or total hip *Z*-score in a group of apprentice and senior flat jockeys. The authors demonstrated that years of race riding did not correlate with either BMD site, suggesting that long-term participation as a jockey and poor BMD values are not associated with low energy availability [23]. However, interpretation of BMD results using the DXA reference population may be the cause of anomalies displayed in the bone health of jockeys such that the significantly smaller size and stature of jockeys is atypical compared to the average European male. Thus, the authors recommended assessing large cohorts of age and weight-matched athletic and non-athletic control subjects [23]. Despite, similar findings observed in a group of Irish flat jockeys [32], a negative relationship between riding experience and bone markers at the femoral neck was reported in NH jockeys. Bone fractures from falls represent the highest percentage of the overall injuries reported by NH jockeys [64], suggesting strategies to prevent fractures and promote bone strength including falls training, exercise workshops and nutrition support are critical for the long-term health and safety of jockeys [32]. Given the serious long-term health implications of low energy availability including complications of cardiovascular, reproductive and skeletal function, and psychological stress further research using detailed assessment criteria is required to establish if low energy availability or other industry associated practices, namely avoidance of weight-bearing exercise are a cause of poor bone health in jockeys [65].

Studies have estimated volumetric BMD or bone mineral apparent density (BMAD) to eliminate the effect of bone size on BMD. Dolan et al. [26] compared the LS BMAD values of jockeys to a group of boxers and active controls. Despite there being no difference between the jockey groups and active controls, the boxer group had a significantly higher LS BMAD, suggesting that BMD differences may be dependent on body size [26]. Similarly, Dunne et al. [32] reported comparative femoral neck and LS BMAD values in flat (0.39 g/cm^3^ vs. 0.39 g/cm^3^ femoral neck and 0.14 g/cm^3^ vs. 0.13 g/cm^3^ LS, [26] and [32], respectively) and NH jockeys (0.38 g/cm^3^ vs. 0.40 g/cm^3^ femoral neck and 0.14 g/cm^3^ vs. 0.12 g/cm^3^ LS, [26] and [32], respectively). However, the LS region used in the calculations by Dolan et al. [26] (L2–L4) was different to that used by Dunne et al. [32] (L1–L4), thus making it difficult to compare results. This is due to advances in DXA software and the region of interest accuracy; hence, the L1–L4 region is now recommended [8]. Apprentice and conditional jockeys displayed lower BMAD values in male jockeys at the femoral neck (0.16 g/cm^3^ apprentice; 0.17 g/cm^3^ conditional) yet similar values at the LS (0.11 g/cm^3^ apprentice; 0.12 g/cm^3^ conditional) compared to previous research of senior jockeys [13]. While the maturation status of the young jockeys was not indicated, some may have been late developers in terms of stage of maturation, and thus, BMD accumulation could have been delayed [13]. Nonetheless, the results are concerning for male apprentice and conditional jockeys as this is happening during a critical stage of bone growth and development, suggesting peak bone mass may not be attained. The consequences of poor bone health in jockeys are likely to have long-term implications on health and performance, many of which are not yet fully understood [33, 34].

There are limited data on female jockeys; however, early research from Leydon and Wall [20] found that 44% of jockeys in New Zealand were classified as osteopenic, six of whom were female jockeys (46%). Further, apprentice jockeys (60%) were identified as having a higher rate of low bone mass compared to senior jockeys (25%). The authors classified low bone mass as a *T*-score <  − 1 at a minimum of two sites. In contrast, Wilson et al. [10] reported female jockeys had a significantly higher *Z* and *T*-score at the total hip and LS than male flat jockeys. The higher BMD in female jockeys could be due to fewer weight-making days such that female jockeys may not engage in severe weight-making practices due to a reduced number of race-rides, lower LM and shorter stature [12]. Similar results were observed by Jackson et al. [13], as female apprentice jockeys were shorter and lighter than male apprentice jockeys. The BMD values at the LS and total hip were significantly higher in female than male jockeys; however, there was no statistical difference at the femoral neck. Lower spinal BMD in male jockeys compared to female jockeys is contrary to the general population [13], while gender differences at the femoral neck are expected as males possess greater bone volume and bone shape at the hip and femoral neck [61]. Currently, 15% of professional jockeys in Ireland are female, an increase of 8% since 2017 suggesting, as female jockey participation continues to increase in horse racing, further research is required to investigate the effects of weight-making practices on the bone health in female jockeys.

To date, longitudinal data evaluating the effects of daily weight-making practices on bone health in jockeys have not been studied. Despite this, previous research has investigated the bone health, disease incidence and health characteristics of retired jockeys from the Ireland and UK [33, 34]. The findings were conflicting, as Mackinnon et al. [34] reported significantly increased odds of osteoporosis in retired jockeys compared to a reference population. However, the study used a standardised core questionnaire to self-report the prevalence of chronic diseases and mental health problems. Furthermore, the reference population (community-dwelling older people) would not have been representative of an athletic population, i.e. retired athletes who had experienced having to make weight, thus providing a comparison based on the impact of weight management [34]. In Ireland, Cullen et al. [33] reported normal BMD *T*-scores for the whole-body, LS (L1–L4) and proximal femur when assessing BMD via DXA scan. The authors concluded by stating the retired jockeys volunteered to take part, such that the participants may represent a healthier sample of the cohort of retired jockeys in Ireland compared to those who did not participate [33]. Therefore, the long-term effects of a weight-restricted lifestyle on bone health from a career in horse racing have not been studied sufficiently to date. Tracking jockeys throughout their career would provide important information about the long-term impact of chronic weight maintenance on bone markers [2, 13].

## Conclusions

This review highlights that the anthropometric measurements and body composition of male jockeys is changing, with a trend for flat and apprentice jockeys being increasingly taller and presenting with higher BF%. Consequently, jockeys that are taller or have higher BF% may be reliant on unhealthy weight loss techniques to reach the low designated riding weights. Conversely, the body mass and height of NH jockeys have remained relatively constant, which in part may be due to the higher weight allowances. Nonetheless, body composition changes in NH jockeys suggest a trend towards increased FM and subsequently increased BF%. The shorter stature and lower LM of female jockeys indicate that making weight is less problematic for them than for male jockeys. However, data are limited in female jockeys, and testing procedures and data reporting have been inconsistent in the jockey literature, including the use of different skinfold equations for predicting BF%. Thus, further investigation is necessary to examine safe and effective weight management strategies for jockeys, such as bespoke nutrition and exercise interventions. The bone health status of jockeys remains unclear due to the different assessment methods, models, sites and classification criteria used to interpret bone measurements. Despite this, male flat jockeys present with consistently lower BMD values at reported sites than male NH and female jockeys. While a number of physical and lifestyle factors in jockeys have been found to influence bone density, namely LM, acute weight management and riding experience, additional research is required to explore the cause of these relationships and the potential long-term effects on bone health from a career in horse racing. Future research must consider implementing a standardised testing protocol using the guidelines set out by ISAK, ISCD, IOF and IFCC to assess body composition, bone density and bone turnover markers. Where possible, a more detailed assessment of bone properties including a volumetric measurement of bone size, strength and geometry using pQCT should be considered. Despite this, access to pQCT equipment may be limited, and thus, BMAD, a volumetric adjustment to approximate the effects of bone depth and body size, could be used. Moreover, the continued development of jockey-specific classification criteria and reference ranges is essential for the appropriate and accurate interpretation of bone health measurements in jockeys.

## Data Availability

Not applicable.

## Abbreviations

BF%: Body fat percentage
BMD: Bone mineral density
BMAD: Bone mass apparent density
CTx: C-terminal telopeptide of type I collagen
DXA: Dual-energy X-ray absorptiometry
FM: Fat mass
fDPD: Free deoxypyridinoline cross-links
NH: National hunt
IFCC: International Federation of Clinical Chemistry and Laboratory Medicine
IOF: International Osteoporosis Foundation
ISCD: International Society for Clinical Densitometry
ISAK: International Society for the Advancement of Kinanthropometry
LM: Lean mass
LMI: Lean mass index
NTx-1: N-telopeptides of type 1 collagen
pQCT: Peripheral Quantitative Computed Tomography
P1NP: Procollagen type 1 amino terminal propeptide
SSI: Strain strength index
